# Continued improvement of cardiovascular mortality in Hungary - impact of increased cardio-metabolic prescriptions

**DOI:** 10.1186/1471-2458-10-422

**Published:** 2010-07-15

**Authors:** Sandor Balogh, Renata Papp, Peter Jozan, Albert Csaszar

**Affiliations:** 1 National Institute of Primary Health Care, (84-88 Jasz Str.), Budapest, (1135), Hungary; 2 Research Centre for Social Studies, Hungarian Academy of Sciences, (30 Orszaghaz Str.), Budapest, (1014), Hungary; 3 2nd Department of Medicine, State Health Centre, (109-111 Podmaniczky Str.), Budapest, (1062), Hungary

## Abstract

**Background:**

During the last 35 years the poor ranking of Hungary on the list of life expectancy at birth among European countries, has not changed. In 1970 our lag behind the leading European countries was the smallest. The gap was growing between 1970 and 1993 but from 1994 onwards the life expectancy at birth in Hungary has increased continuously and somewhat faster than in other European countries. The aim of this study was to analyze the association between decreasing cardiovascular mortality rates, as a main cause of death and the increase in cardio-metabolic prescriptions and possible changes in lifestyle behavior.

**Methods:**

Analyses were conducted on national data concerning cardiovascular mortality and the number of cardio-metabolic drug prescription per capita. The association between yearly rates of cardiovascular events and changes in antihypertensive, antilipidemic and antidiabetic prescription rates was analyzed. The changes in other cardiovascular risk factors, like lifestyle were also considered.

**Results:**

We observed a remarkable decline of mortality due to stroke and acute myocardial infarction (AMI). The fall was significantly associated with all prescription rates. The proportion of each treatment type responsible for suppression of specific mortality rates is different. All treatment types comparably improved stroke mortality, while antilipidemic therapy improved AMI outcome.

**Conclusions:**

These results emphasize the importance of a comprehensive strategy that maximizes the population coverage of effective treatments. Hungary appears to be at the beginning of the fourth stage of epidemiologic transition, i.e. it has entered the stage of delayed chronic noninfectious diseases.

## Background

The poor ranking of Hungary on the list of life expectancy at birth in the European Union (EU-25) (22^nd^ position in 2005 in the EU-25 countries) has not changed during the last 35 years but the size of deviation - expressed in years - from other countries has changed substantially (Table 1).

**Table 1:** Life expectancy at birth (years) (1970-2005)^1^

	1970	1980	1990	1993	2000	2005
**Austria**	70.02	72.78	75.99	76.47	78.66	79.70

**Denmark**	73.51	74.24	75.11	75.37	77.22	78.44

**Finland**	70.40	73.73	75.13	76.75	77.78	79.36

**France**	72.93	74.91	77.62	78.71	79.35	80.49

**Hungary**	69.30	69.12	69.45	69.17	71.93	73.02

**Italy**	71.66	74.38	77.20	77.71	79.75	80.30

**Spain**	72.88	75.60	77.00	77.75	79.49	80.44

**Sweden**	74.83	75.87	77.77	78.33	79.92	80.82

**United Kingdom**	71.95	73.71	75.92	76.30	78.06	79.29

In 1970 our lag behind the leading European countries was the smallest, specifically 5.53 years compared to Sweden, which was at the top of the list for life expectancy at birth (Table 2).

**Table 2:** Difference in life expectancy at birth (1970-2005) between Hungary and Sweden, Denmark, EU-15 countries respectively (SWE = Sweden, DEN = Denmark)^1^

	1970	1980	1990	1993	2000	2005
**SWE-HUN**	5.53	6.75	8.32	9.16	7.99	7.80

**DEN-HUN**	4.21	5.12	5.66	6.20	5.29	5.42

**EU-15-HUN**	2,53	5.06	7.03	7.83	6.83	6.91

The gap was growing between 1970 and 1993 i.e. in 1993 the difference was 9.16 years compared to Sweden and 6.2 years compared to Denmark, which ranked just one position ahead of Hungary (in 1993 EU-15 countries) [1]. From 1994 the life expectancy at birth in Hungary has increased continuously and somewhat faster than in other European countries. More recently (in 2005), the differences to Sweden and Denmark were 7.8 and 5.42 years, respectively.

**Figure 1:**
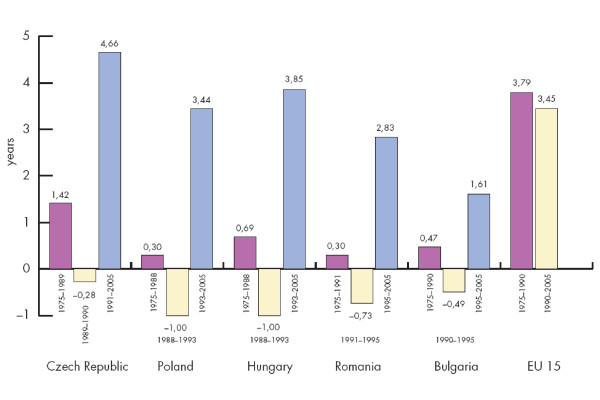
Changes in life expectancy at birth in some Central- and Eastern-European and EU-15 countries in the period 1975-2005^1^

Figure 1 summarizes the changes of life expectancy at birth in five Central- and Eastern-European (CEE-5) and EU-15 countries before and after the post-communist political transition. Overall, longevity increased by 0.14 - 3.24 years in Central- and Eastern-Europe. After the transition, life expectancies temporarily decreased in Central- and Eastern-Europe by 0.3 - 1.0 year but later improved by 1.61 - 4.66 years. The periods with decrease and increase in longevity were slightly different in the countries examined.

**Figure 2:**
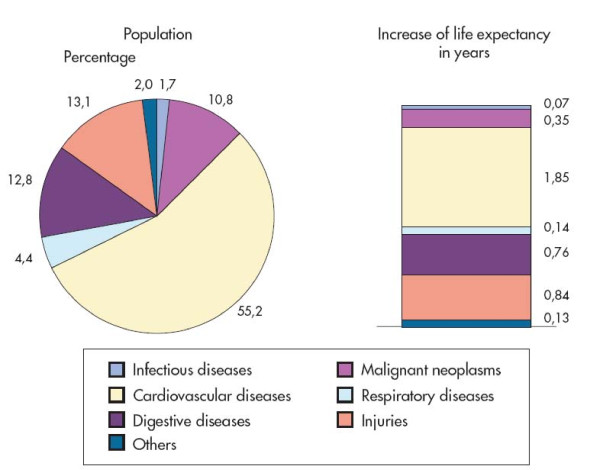
**Contribution of the main causes of death to the decrease of mortality rate and to the increase in life expectancy at birth between 1993-2000 in Hungary**^2^

In Central- and Eastern-European countries, including Hungary, cardiovascular diseases are the leading cause of total mortality, cardiovascular mortality representing 50-60 percent of the total mortality [2]. The new epidemiological period (1993-2005) is characterized mainly by a decline in the rate of cardiovascular mortality, which is responsible for a 55,2% reduction of total mortality in Hungary [2]. The cardiovascular mortality rate was 640.48/100 000 inhabitants in 1993 and 460.25/100 000 in 2006, respectively, which represents a 28.1% decrease [3]. On the contrary, mortality caused by malignancies has not changed substantially. Figure 2 shows the contribution of the main causes of death to the decrease of mortality rate and to the increase in life expectancy at birth [2].

The most impressive component of the reduced cardiovascular mortality rate is the decreased number of fatal acute myocardial infarctions (AMI). In 2007 the mortality due to AMI dropped to 8400 from 15000 in 1993 [4]. The cardiovascular mortality rate per 100 000 inhabitants decreased by 53% during this period. Since 1970 mortality due to stroke has declined by 54.5% and this is associated with an increase in high blood pressure therapeutic prescriptions [2]. The improvements in AMI- and stroke-mortality rates have significantly contributed to the total increase of life expectancy at birth (0.68 and 0.80 years, respectively).

**Figure 3:**
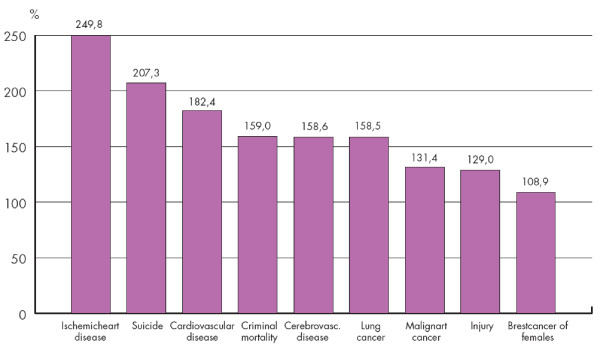
**Ratio of the mortality rate by main cause of death in Hungary compared to EU-25 countries**^1^

Despite the recent improvement of life expectancy at birth in Hungary, the absolute value remains low compared to the international data (the difference was 6.91 years in 2005). This situation might be explained by the unusually high mortality rates of some important diseases in Hungary compared to EU-25 countries [1] (Figure 3).

'Avoidable Mortality' through health care intervention is another technique used to quantify the public health status of a country [5]. In the period 1993-2006 mortality avoidable by medical management lowered by 43% in Hungary, and expressed as a proportion of total mortality, it has been reduced from 22% to 16% [2]. With regard to medical intervention, approximately half of the cardiovascular mortality rate fall was attributable to medical therapies against myocardial infarction, heart failure and angina pectoris.

In spite of some limitations, the IMPACT model is primarily used [6] to estimate the number of cardiovascular heart disease (CHD) deaths prevented by each specific cardiac intervention, or risk factor decline. The comparison between the increase of cardio-metabolic prescriptions and cardiovascular mortality rate represents another approach applied to obtain a more precise estimate of the role of the reduction of each risk factor. One of the purposes of our analysis was to examine this association in the period 2000-2008 in Hungary.

## Methods

The Hungarian mortality database (Hungarian Central Statistics Office, CSO) was the source of data on mortality and the National Health Insurance Fund provided the number of prescriptions per capita, both at population level. Both databases are freely accessible; no specific approval was required for use. The yearly data from 2000 until 2008 were used to examine the association between changes in antihypertensive, antilipidemic and antidiabetic prescription rates and changes in cardiovascular event rates. Regarding other cardiovascular risk factors, like lifestyle behavior: diet, smoking, alcohol consumption, the CSO data on consumption of fat-, tobacco- and alcohol per capita were used.

### Death from Stroke, Acute Myocardial Infarction

Age-adjusted mortality rates from stroke and acute myocardial infarction among individuals aged >20 years were considered from the Hungarian Mortality database. Diagnoses were defined according to the International Classification of Diseases Ninth and Tenth Revisions (ICD-9, ICD-10). Mortality rates were standardized to the age (in groups 25-49, 50-64, 65-79 and 80-years of age) and gender distribution of the Hungarian population aged ≥20 years in the year 2000. The hemorrhagic and ischemic stroke data were included. The data source was the reports of the in-patient care services to the National Health Insurance Fund about the hospitalized patients with main diagnosis classified as I60-I62; I63-I66 according to the ICD.

### Intervention: Antihypertensive, Antilipidemic and Antidiabetic Therapy

Information about antihypertensive, antilipidemic and antidiabetic prescriptions was obtained from the National Health Insurance Fund database compiled yearly from the dispensing records of all retail pharmacies from the country. This database does not include information on physician characteristics, patient-level data (e.g., age, gender, co-morbidities, or concomitant medications dispensed), or indications for use. The patient was considered as treated if the medicine was taken for at least 6 months in the respective year. We examined the number of prescriptions dispensed between 2000 and 2008 and normalized the age to the population of Hungary that was over the age of 20 years in the year 2000. Data were not standardized according to gender. Regarding the treatment patterns related to the ATC groups, no deeper analysis was performed. This survey had no population wide coverage and data shows a cross-sectional state, no yearly changes. The number of Percutaneous Transluminal Coronary Angioplasty (PTCA) and coronary bypass interventions were examined. The National Health Insurance Fund data were available for 2004-2007 only.

### Statistical Analysis

The relationship between standardized yearly rates of mortality per 10 000 inhabitants and the numbers of yearly prescriptions per 10 000 inhabitants was examined. This analysis was designed to assess the association between the changes in prescriptions and the changes in cardiovascular mortality rates as a function of time (years). In each case, a regression analysis was performed for the standardized mortality rate against prescription rates for hypertension, hyperlipidemia and diabetes, respectively. We also determined the correlation coefficients between prescriptions and mortality rates. All of the computations were solved using MedCalc statistical software.

## Results

**Figure 4:**
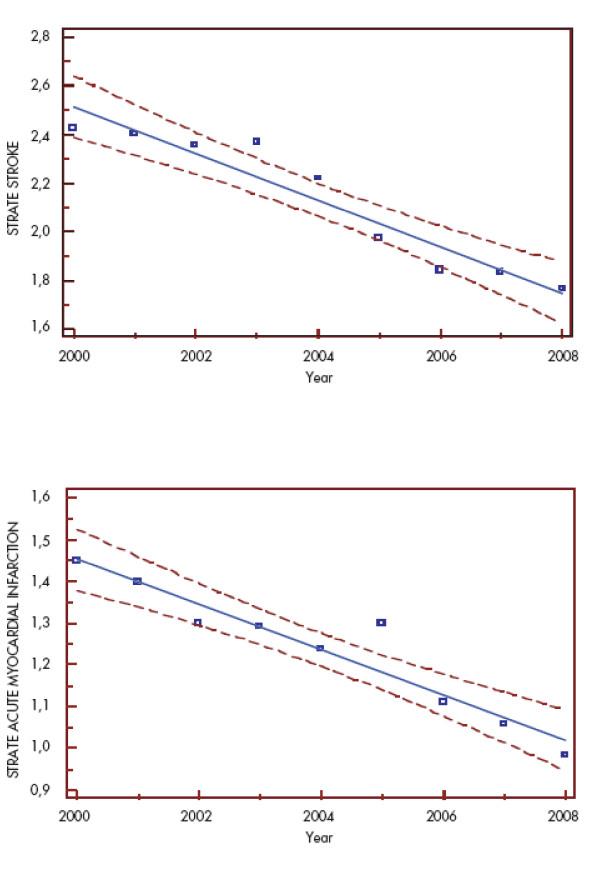
**Decrease in stroke and acute myocardial infarction age and gender standardized mortality rate between 2000-2008 in Hungary**^3^

In Hungary there were 36.4 and 23.6 fewer deaths/per 1000 deaths from stroke and acute myocardial infarction in 2008 than in 2000, respectively. The age and gender standardized annual mortality events improved significantly for stroke and myocardial infarction (Figure 4).

Among hospitalized patients, the number of those with a hemorrhagic stroke decreased by 86% in the period 2000-2008. The percentage of patients hospitalized and cured with ischemic stroke almost doubled during the nine years. The hospital mortality for both types of stroke decreased continuously over the years: 84% for hemorrhagic stroke and 55% for ischemic stroke. Regarding cardiac surgical interventions, the coronary bypass operations decreased by 75% and the PTCAs multiplied 1.5 times.

The per capita consumption of different types of nutrients showed slight changes during 2000-2007. The fat quantity was the same over the eight years: 145,5 + 3,2 g/day/per capita. According to the COICOP (Classification of Individual Consumption According to Purpose) the ratio of spending on tobacco increased year by year, in total with 122.3% from 2000 till 2007; the spending on alcohol increased from 3.7% to 4.4% [4]. No significant change in consumption of fruits and vegetables occurred.

**Figure 5:**
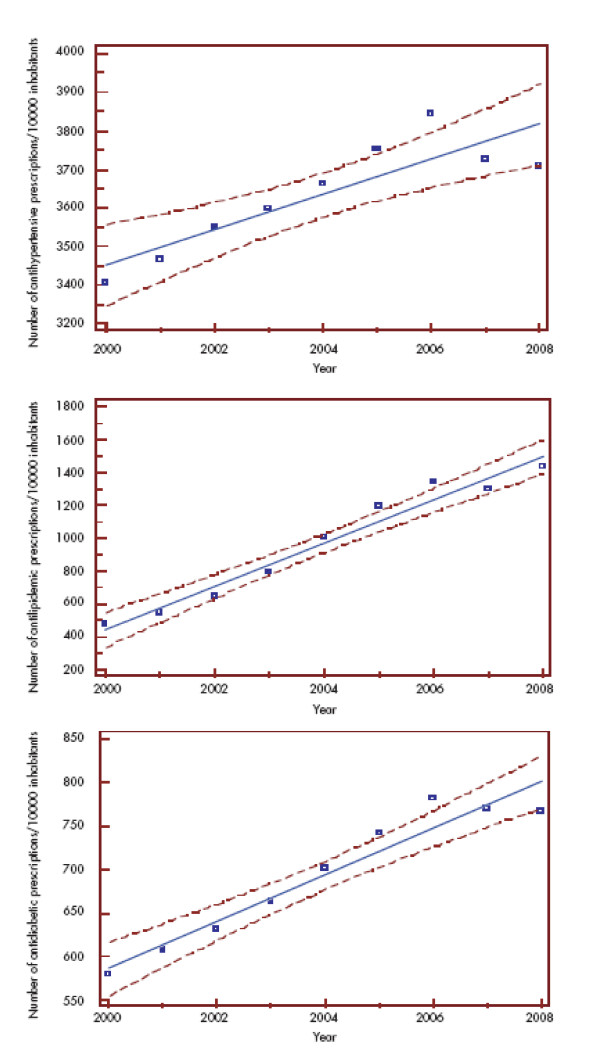
**The increase of annual prescriptions rate per 10 000 inhabitant for antihypertensive (A), antilipidemic (B) and antidiabetic (C) drugs between 2000 and 2008 in Hungary**^4^

Total annual antihypertensive, antidiabetic and antilipidemic drug prescriptions increased by 110, 134 and 306% between 2000 and 2008, respectively [7]. The absolute number of prescriptions in 2008 was the highest for antihypertensive drugs followed by antilipidemic and antidiabetic drugs. The annual growth was significant for each prescription (Figure 5).

The declines of mortality by stroke and AMI were significantly associated with all prescription rates (Table 3). The correlation between specific mortality rates and specific treatments differed. The highest correlations were observed between stroke mortality and all treatment types and between AMI mortality and antilipidemic therapy.

**Table 3:** Correlations between prescriptions and mortality rates in the period of 2000-2008 in Hungary

		Stroke mortality/Population	Acute myocardial infarction/Population	Total mortality/Population
Number of antidiabetic prescriptions/Population	Correlation Coefficient	-0.945	-0.874	-0.037
	Significance Level p*	<0.001	0.002	0.926
	n	9	9	9

Number of antilipidemic prescriptions/Population	Correlation Coefficient	-0.963	-0.892	-0.070
	Significance Level p*	<0.001	0.001	0.859
	n	9	9	9

Number of antihypertensive prescriptions/Population	Correlation Coefficient	-0.888	-0.802	0.016
	Significance Level p*	0.001	0.009	0.967
	n	9	9	9

*p: Pearson correlation coefficient

## Discussion and Conclusions

In developed countries, cardiovascular diseases are the leading cause of mortality and hospitalization, and the major contributors to healthcare costs [8-13]. The decline of cardiovascular mortality rate is a great success in many countries, including Hungary. In the introduction we summarized the continuous improvement in cardiovascular mortality rate in Hungary.

Often the numbers of CHD deaths prevented or postponed by each specific cardiac intervention and by each risk factor change are calculated using the IMPACT mortality model [6]. This model includes the number of CHD deaths, the specific medical and surgical treatments and population-trends in major cardiovascular risk factors. In the frame of the IMPACT model, the mortality reduction credited to each treatment is calculated using the published effectiveness of cardiovascular treatments and risk factor reductions obtained from meta-analyses and controlled trials [6,14-16]. Explanations for the mortality decreases remain controversial [17]. Many authors credit the increasingly widespread use of effective therapies such as thrombolysis, aspirin, ACE inhibitors, statins, and coronary artery bypass surgery [18,19]. Others highlight reductions in major cardiovascular risk factors such as high blood cholesterol, smoking and high blood pressure [17,20].

The majority of authors suggest that risk factor improvements explain more of the mortality decline than do treatments. For example, it has been estimated that the proportion of mortality decline attributable to risk factor reductions was 57% in the United States between 1980 and 1990 [21]; 60% in Auckland, New Zealand, between 1974 and 1981 [22] and 52% between 1982 and 1993 [6]; and 60% in Scotland between 1975 and 1994 [23]. In Finland between 1982 and 1997 CHD mortality rate declined by 63% and the findings highlight the value of risk factor reduction which explains 52.3-72% of the given improvements [24].

These modeling studies have some caveats: they are dependent on the variable quality and extent of available data on cardiovascular risk factor trends and CHD treatment uptakes [25]. All require numerous data inputs constrained by selection bias and/or make explicit assumptions. However, this general approach merits further investigation. It is necessary to develop more simplified methods to identify the most important health care activities required to improve mortality and morbidity rates. This paper describes one such activity: an increase in special medical prescriptions. Significant decreases in cardiovascular events initially followed the introduction and use of antihypertensive drugs in the 1970s [21,26-28]. Despite robust randomized trial evidence confirming the benefits of lowering blood pressure with antihypertensive agents, the expected benefits of antihypertensive treatment in reducing cardiovascular events have not been seen in several observational studies of usual clinical practice [18-20,29]. The first published statistical study which successfully associates increased antihypertensive therapy with decreased cardiovascular disease on a national scale, and the improvement in outcomes with the start of a national hypertension management effort, was published in early 2009 [30]. This study prompted our current analysis, which takes into account a broader range of cardiovascular treatment modalities.

Complex and multiple causes lead to the Hungarian changes of the last decade presented in this article. Half of the 4.14 years gained in life expectancy is due to the decline in cardiovascular mortality. Our findings support the role of antihypertensive treatment in improving cardiovascular mortality rates. The antihypertensive prescriptions show the highest absolute number during the years. However, records show that the antilipidemic therapies increased most, exceeding the total number of prescribed therapies for diabetes in 2008. We analyzed the antilipidemic and antidiabetic prescription rates to address whether the same association exists for these classes of drugs. We report here that the extension of cardiovascular treatment modalities has a beneficial influence on cardiovascular events. In order to prove this proposition and to reveal the role of other risk factors we have to reflect on the changes regarding: lifestyle, health care services management, reach of therapeutic targets.

The increasing awareness of the population towards healthy behavior is associated with the positive results in health care statistics, such as decreased mortality, longer life expectancy. However, healthy behavior is not characteristic of the Hungarian population. According to the CSO data, presented in the results, tobacco consumption increased, there was no positive change in fat consumption, and a constant fruit and vegetable usage complete the picture of an unhealthy way of life. Unfortunately, we could not find data about physical activity set over time. The data from 2000, 2003 show that the population over 35 forgets him/herself in the armchair [4].

The period following 1990 was characterized by large changes in the health care system, including financing. In spite of geographical differences, all the modern medicines, new technologies and interventions were made available for the population. The institutional background of the interventional cardiology developed. We note here, that the data about hospital interventions and in-patient care could be biased by financial issues, as the reimbursement is related to the medical care according to disease related groups (DRG).

The great change in the way of life has not been done, the hospital interventions are at the very end of the management of CHD, but the preventive approach in the field of general practice/family medicine brings the changes in outcomes. The number of patients undergoing cardio-metabolic treatment increased. Apart from the number of those under pharmaceutical treatment, data about the treatment targets of blood pressure and blood lipids were get from survey performed by Hungarian Society of Hypertension [31]. This survey had no population wide coverage and data shows a cross-sectional state, no yearly changes. According to the study, in 2005 39.3% of hypertensive patients under treatment had blood pressure lower then 140/90 mmHg. This ratio of the "well treated" (blood pressure under 140/90 Hgmm) group was 44.6%. The education for primary health care professionals led to better results in the chronic treatment.

These results emphasize the importance of a comprehensive strategy that maximizes the population coverage of effective treatments and actively promotes a prevention program. There are several limitations to the analyses that we have conducted. Underlying cardiovascular risk factors of the Hungarian population are likely to have increased (obesity, diabetes mellitus), there were no data on changes in physical activity over time and the data about the increase on tobacco spending are soft, includes the price increase too.

There was also a considerable increment in aspirin use. The role of acute coronary interventions has also grown - the increase was 49.1% between 2004 and 2007 in Hungary. Health promotion and preventive and curative medicine have contributed to the improving CHD epidemiological situation. Hungary appears to be at the beginning of the fourth stage of epidemiologic transition, i.e. having left the stage of chronic noninfectious diseases; it has entered the stage of delayed chronic noninfectious diseases.

## Competing interests

The authors declare that they have no competing interests.

## Authors' contributions

SB has been involved in drafting the manuscript and revising it critically for important intellectual content. RP has been involved in the acquisition of data, analysis and interpretation of data. PJ has made substantial contributions to conception, acquisition of the data on causes of mortality and their contribution to the favorable changes in life expectancy. ACs has made substantial contributions to conception and design, has given final approval of the version to be published. All authors read and approved the final manuscript.

## Pre-publication history

The pre-publication history for this paper can be accessed here:

http://www.biomedcentral.com/1471-2458/10/422/prepub
